# Non-concussive impacts, cognition, and the menstrual cycle: a proof-of-concept study of elite female footballers

**DOI:** 10.1007/s00221-026-07300-2

**Published:** 2026-05-21

**Authors:** Benjamin Tari, Megan Lowery, Chris M. Jones, Kieran Austin, Mike Loosemore, Paul W. Burgess, Flaminia Ronca

**Affiliations:** 1 Institute of Sport, Exercise and Health, University College London, London, UK; 2 Institute of Cognitive Neuroscience, University College London, London, UK; 3 Sport and Wellbeing Analytics Limited, Swansea, UK; 4 Institute of Applied Sciences, University of Chichester, Chichester, UK

**Keywords:** Heading, Mouthguard, Accelerometery, Luteal

## Abstract

Evidence indicates females may be more susceptible to sports-related concussion with worse and prolonged symptom severity according to menstrual cycle phase. We investigated whether menstrual cycle phases influence non-concussive heading kinematics in elite female footballers, and whether these impacts affect an athlete’s cognition. Five eumenorrheic elite female footballers (M_age_ = 23 ± 4 years) participated in a 16-week proof-of-concept study wherein menstrual cycle phases were tracked, and cognition was monitored. Participants performed a weekly controlled heading drill by heading a ball thrown from 5 m away. Head accelerations were measured using custom-moulded PROTECHT instrumented mouthguards. 256 headers revealed no significant differences in head acceleration across the various phases of the menstrual cycle. However, change in cognitive performance was related to heading completion and menstrual phase. Hormonal fluctuations during the menstrual cycle may influence cognition independent of head impact biomechanics among elite female athletes, under controlled conditions. We note that our study also demonstrated the safety and efficacy of the mouthguard equipment employed here, as well as the ease with which the protocol was received by the athletes. These outcomes should be considered when implementing future research with larger cohorts and the inclusion of match-related heading.

## Introduction

Association Football (football/soccer) is one of few sports where the head can be intentionally used to direct the path of the ball during play (Spiotta et al. 2012), and where the athlete undertakes a voluntary non-concussive head impact (Sarajärvi et al. 2020; Weber et al. 2023). A concussion is defined as a pathophysiological process which affects the brain via some biomechanical force (e.g., rapid and sudden accelerations) (McCrory et al. 2017) and differs from a non-concussive impact in that the latter does not result in observable concussive symptoms (Bailes et al. 2013). Repetitive head impacts, both concussive and non-concussive, have been identified as a risk factor for neurodegenerative disease (Baugh et al. 2012; Nowinski et al. 2022). National governing bodies (NGBs) have recognised the potential danger of heading, with multiple NGBs implementing heading guidelines for youth players and professional athletes (Football Association, n.d.). The removal of heading from training, and the game itself, for youth players in the UK began in 2024–25 (Football Association, n.d.-b; *New FA Rule to Ban Headers in Youth Football Games*, 2024), and this represents a seismic shift for the adult game. Therefore, alternative solutions that identify and help to understand the risk factors of head accelerations and associated injuries are required.

Non-concussive head impacts have been shown to trigger cortical dysfunction in athletes absent of symptom presentation (Broglio et al. 2012; Wallace et al. 2018; Nowak et al. 2020; Zuidema et al. 2024), and cohorts of former footballers have reported a greater risk of developing neurodegenerative diseases (Mackay et al. 2019; Ueda et al. 2023). Furthermore, some studies have observed an association between exposure to repetitive heading and the appearance of neuropsychological impairment (Matser et al. 1998; Downs and Abwender 2002; Rutherford et al. 2005; Lipton et al. 2013; Levitch et al. 2018; Bruno and Rutherford 2022), whereas others report no such association (Janda et al. 2002; Straume-Naesheim et al. 2005; Kemp et al. 2016; Kenny et al. 2019; Huber et al. 2023). These conflicting findings are largely due to the heterogeneity of populations tested, and the methods implemented including retrospective self-report measures of heading exposure. Female footballers have reported more rapid head accelerations than males during controlled heading scenarios (Caccese et al. 2018). It has also been reported that female footballers may be at a heightened risk of adverse neurological outcomes compared to their male counterparts following repetitive heading (Rubin et al. 2018). Notably, however, the mechanisms that underlie these sex differences are not yet understood (Rubin et al. 2018; Prien et al. 2020; Cente et al. 2025). Poorer neck strength in females (Caccese et al. 2018; Peek et al. 2021) and an association between the menstrual cycle phase and increased prevalence of SRC have been previously cited (La Fountaine et al. 2019).^1^ Although more quantitative research is required (Martínez-Fortuny et al. 2023), preliminary evidence suggests a relationship between injury likelihood (e.g., SRC) and hormone concentrations across the menstrual cycle. In addition to functional and anatomical brain changes, SRCs and repetitive heading have been shown to influence cognitive functioning (Taylor et al. 2018; Collins et al. 2023; Patel et al. 2024). It is therefore prudent to understand the effects of hormone fluctuation on cognition. Research suggests a relationship between various cognitive domains and menstrual phase, with worse spatial cognition often reported in the ovulatory (i.e., peak oestrogen) and luteal (i.e., elevated oestrogen and progesterone) phases, and longer reaction times (RTs) observed in the luteal phase (Ronca et al. 2025). Potential mechanisms underpinning these differences may involve changes in oestrogen and progesterone levels which have excitatory and inhibitory effects on the cerebral cortex, respectively (Shaywitz et al. 1999; Smith et al. 1999, 2002). Taken together, the above suggests the need to better study how menstrual-phase-related changes to cognitive function might be exacerbated by repetitive non-concussive head injury and whether these factors interact in a bi-directional manner. This proof-of-concept study aims to quantify and describe the immediate and short-term effects of repetitive non-concussive head impacts on (1) head acceleration metrics and (2) cognitive performance in female footballers across the menstrual cycle, and to determine best practice for future “real-world” studies. We will define and quantify tangible outcomes including the feasibility of using mouth-guard-based technology to provide personalised monitoring of head acceleration in elite female footballers, as well as whether cognitive function might be influenced by changes in menstrual cycle phase. We expected that headers would have the greatest negative impact on head acceleration (i.e., faster) and cognitive performance (i.e., more errors, longer RT) during the luteal phase (high progesterone), and heading may engender fewer adverse cognitive outcomes during ovulation (high oestrogen). This may be due to fluctuating hormone concentrations concomitant with a supposed “window of vulnerability” wherein head and body injury prevalence increases (Chidi-Ogbolu and Baar 2018; La Fountaine et al. 2019; Barlow et al. 2024).

## Materials and methods

### Participants

Nine elite (Tier 4 according to the McKay framework; McKay et al. 2022) female footballers were recruited for this proof-of-concept study during pre- and early-season training and five (M_age_ = 23 ± 4 years) completed the entire study protocol. Participants belonged to a single club competing in the third tier of English women’s football in England, and were recruited as part of a convenience sample, primarily due to the practical demands of recruiting both within elite sport and within a naturally menstruating population. Participants were included if they reported a regular cycle of between 28 and 35 days, had not recently suffered an SRC within the last 6 months, had self-reported absence of regular spotting outside of menstruation, and had tracked their period for at least three months prior to beginning the study. Ethical approval was granted by the University College London Research Ethics Committee (13985/007) and participants provided informed written consent prior to any study activity. Research was carried out in line with Consensus Head Acceleration Measurement Practices (CHAMP) recommendations (Arbogast et al. 2022, 2023), and according to the most recent iteration of the declaration of Helsinki with the exception that participants were not included in a database.

### Apparatus and procedure

#### Cycle tracking

Participants were asked to provide calendar-based counting of their menstrual cycle for 16 weeks and to provide a self-reported indication of the day of their menstrual cycle (e.g., day of bleeding and flow intensity) and associated symptoms (e.g., menstrual cramps, headaches, mood changes) through the FitrWoman (Brown 2018) application. The use of hormonal contraceptives prior to or during the study was not considered or recorded. Notably, participants were recruited to the study at the same time and thus began testing at different phases of their cycle. Participants were provided urinary ovulation tests (Clearblue Advanced Digital Ovulation Test, Swiss Precision Diagnostics GmbH, SPD, Petit-Lancy, Switzerland) to confirm a surge in luteinizing hormone and provide an estimate of ovulation onset. Ovulation testing began on or around day nine following cessation of bleed and continued until ovulation was detected. Once a positive test occurred, participants recorded this using the FitrWoman application. The menstrual cycle phase was considered in five parts: menstruation; late follicular; ovulation; mid luteal; and late luteal phases.^2^ Menstruation was reported via self-logging in the FitrWoman application. Late follicular phase was estimated by the end of bleeding and before the onset of positive ovulation. Mid luteal phase was characterised from the day following ovulation until five days before the next menstruation phase; these last five days were considered the late luteal phase.

#### Measures of head acceleration

Prior to completing any study protocols, participants were given a PROTECHT mouthguard system (Version 2; Sport and Wellbeing Analytics, Swansea, United Kingdom) fitted with a tri-axial accelerometer (± 400* g* range at 12-bit resolution; H3LIS331DL, STMicroelectronics, Geneva, Switzerland) and a tri-axial gyroscope (± 35 rad*/s* at a 12-bit resolution LSM9DS1, STMicroelectronics, Geneva, Switzerland) sampling at 1 kHz. Mouthguards were custom moulded to individuals’ teeth using a boil-and-bite impression, which were then modelled and printed using computer-aided design (CAD) software. The PROTECHT system has been previously validated to provide reliable measures of peak linear acceleration (a_linear_), peak rotational velocity (v_rotational_), and peak rotational acceleration (a_rotational_) resulting from impacts up to 171 g*,* 40 rads/s, and 10 krads/s^2^, respectively, as well as the estimation of tissue-level injury metrics (Jones et al. 2023; Austin et al. 2023). Testing revealed that some headers might engender linear resultant accelerations below the generally accepted 10 g cut-off for impact classification (King et al. 2015). Accordingly, we chose to adopt a 5 g threshold. Data were continuously collected while participants were fitted with the mouthguards, and heading trials which exceeded this threshold triggered a 104 ms data sampling window (i.e., − 10 ms pre-trigger to 94 ms post-trigger).

#### Heading protocol

**Fig. 1:**
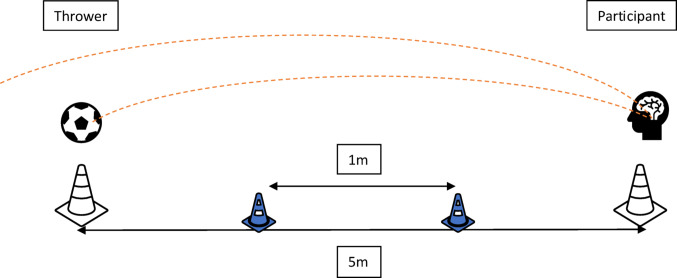
A schematic representation of the heading drill employed in this study protocol. The blue cones spaced 1 m apart represent the calibration objects for football trajectory calculations

Participants were required to perform a controlled heading drill before their normally scheduled training session with a researcher. In total six heading drills were completed over the course of the 16-week trial. The selected heading drill was designed to be reflective of typical heading seen within training and was based on a protocol designed by Peek and colleagues (2021). Footballs were size-5 and inflated in line with FA regulations (8.5–15.6 psi) (Shewchenko et al. 2005a). Participants and the researcher stood 5 m apart, and researchers threw a regulation football toward participants. Participants were required to execute 10 defensive headers whilst wearing their instrumented mouthguard (see Fig. 1). A defensive header was defined as aiming to head the ball back and over the head of the researcher, aiming for ‘maximum distance'. Ball velocity was measured using two-dimensional motion capture via a camera (Sony CX240E, Sony Group Corporation, Tokyo, Japan) that was affixed atop a rigid tripod 10 m away from, and perpendicular to, the participant and researcher and sampling at 30 frames per second. A calibration object was set on the floor between the participant and researcher directly below the trajectory of the thrown football and spaced 1 m apart. In an attempt to standardise velocity, researchers were instructed to propel the ball in a similar manner and were trained by senior researchers prior to the study period. Total testing time was between 10 and 15 min per session. All mouthguard impacts were video verified after aligning “video time” with mouthguard timestamps.

#### Measures of cognition

Cognitive testing was carried out by members of the study team (including sport exercise interns) who were appropriately familiarised with the study protocol and trained in test delivery. Participants completed cognitive testing immediately before and after the heading drill at each of the six testing sessions. When possible, testing was completed at the same time of day and in the same training area as per their scheduled training sessions. Because of the latter, spacing of sessions was not necessarily scheduled according to menstrual phase. The cognitive battery was previously developed to assess sport-specific cognition and validated by our lab in the context of measuring menstrual cycle effects (Ronca et al. 2025), and included simple reaction time, sustained attention, inhibition, and spatial timing anticipation. We provide a brief overview of the cognitive battery below but encourage the reader to review Ronca et al. (2025) and Watson et al. (2024) for additional information.

In the simple reaction time task, participants were instructed to press the spacebar as quickly as possible when a smiling or winking face appeared at the centre of their screen. Simple reaction time was included in the battery due to previous research that reports a relationship between RT and injury risk (Swanik et al. 2007; Wilkerson 2012).

The sustained attention task was administered after the simple reaction time task and consisted of a No-Go/Go task with extended intervals. Participants were instructed to press the spacebar when they saw a winking, but not a smiling face (i.e., a Go trial). Sustained attention was included in the battery given its sporting relevance for focusing on a task at hand while preparing to execute a response (e.g. being ready to defend a goal kick or a punch).

The inhibition task consisted of a Go/No-Go task, which used the same stimuli as the sustained attention and simple reaction time tasks, and was always administered following these tasks. In contrast to the sustained attention task, participants were instructed to press the spacebar when they saw a smiling but not a winking face. Inhibition was included in the battery due to its relevance toward restraint of an action in fast-paced situations (e.g. changing defending technique when an opponent is in the penalty area).

Finally, the spatial timing anticipation task consisted of images of two footballs moving towards each other at a constant speed on a horizontal line from opposite ends of the screen. Movement speeds ranged from 6 to 18 m/s (i.e., similar to those observed during gameplay) (Isokawa and Lees 1988; Shewchenko et al. 2005b) and for half the trials, the two objects moved at the same speed. Participants were instructed to press the spacebar when they believed the two objects would collide. This task was included due to its relevance in determining the positioning of two moving objects in time and space at different speeds. The ability to anticipate the position of an object in space and time is likely to be highly relevant to performance and injury occurrence in sport (e.g. when timing the interaction of one’s limb, a ball and other players).

### Dependent variables and statistical analysis

Mean RTs were calculated for the former three tasks after removing trials with RT > 125 ms. A Composite Reaction Time was then calculated using these values and is included in our analysis. All incorrect responses (i.e., sum of incorrect No/Go responses, ITI presses, responses with RT > 125) were counted as commission errors for the purpose of this analysis. Intra-individual variability of RT was calculated as the standard deviation of incorrect RT trials. Mean timing error was calculated as the mean absolute difference in ms between the participant button press and the actual collision time. Intra-individual variability of timing error was defined as the standard deviation of timing error.

Ball motion was analysed within Kinovea 2023.1 (Kinovea, Open-Source Software) to ascertain ball velocity in the 5 video frames before head contact. Mouthguard linear acceleration and rotational velocity time-series data were directly measured from the tri-axial accelerometer and gyroscope (see Table 1 for variable definitions). Raw kinematic data were filtered using a 4th-order low-pass Butterworth filter with a cut-off frequency of 160 Hz (Jones et al. 2023). Rotational accelerations were derived from the filtered rotational velocity time series using a 5-point stencil derivative and re-filtered using the same Butterworth filter as above. Filtered linear acceleration as transformed to the head centre of mass utilising the relative acceleration equation (Tooby et al. 2024). Peak values were defined as the maximum numerical value of the vector norm in a time-series. All kinematic data analysis was conducted via a custom MATLAB script (MATLAB v2023a, The Mathworks, Natick, Massachusetts, United States of America).

**Table 1:** A representation of head acceleration variables and associated definitions

Variable	Definition
Total impact (n)	Number of impacts recorded
Peak linear acceleration (g)	The mean peak resultant linear acceleration value attained for all impacts
Peak rotational acceleration (rad/s^2^)	The mean peak resultant rotational acceleration value attained for all impacts
Max linear acceleration (g)	The highest resultant linear acceleration value attained for all impacts
Max rotational acceleration (rad/s^2^)	The highest resultant rotational acceleration value attained for all impacts
Contact load (AU)	The sum of z-scored linear and rotational accelerations

Statistical analyses were performed using R Studio (Posit Team 2025). Linear acceleration and rotational acceleration data were skewed rightward and were therefore log-transformed. A linear mixed model was used to identify differences in individual impact metrics across 256 separate heading events between the five menstrual cycle phases (i.e., menstruation, late follicular, ovulation, mid luteal, and late-luteal). Fixed effects and interactions were performed with Bonferroni post-hoc tests to identify pairwise differences. Composite Reaction Time, intra-individual variability of RT, as well as timing error and commission error data were analysed via a two (i.e., time; before headers, after headers) by four (i.e., phase; menstruation, late follicular, ovulation, mid luteal) repeated measures ANOVA because not enough participants were able to provide data during the late-luteal phase. There were no other missing data. Pairwise post hoc comparisons with Holm correction were conducted when the data was not normally distributed. Cognitive variables were BoxCox transformed to achieve normality. Linear mixed models with random effects were employed to investigate whether head acceleration predicts changes in cognitive function. Statistical significance was achieved when *p* < 0.05.

## Results

### Participants

Nine elite female footballers were recruited from a single elite football team. Of these, five completed testing at least four times (age = 23 ± 4 years; M_height_ = 163.5 ± 5.5 cm, M_body mass_ = 62.4 ± 6.2 kg, average cycle length = 26 ± 2 days). Four participants dropped out of the study due to injury (n = 1) and personal commitments (n = 3). These athletes’ menstrual cycles were tracked between 9th August 2023 to 2nd December 2023.

### Head accelerations

**Fig. 2:**
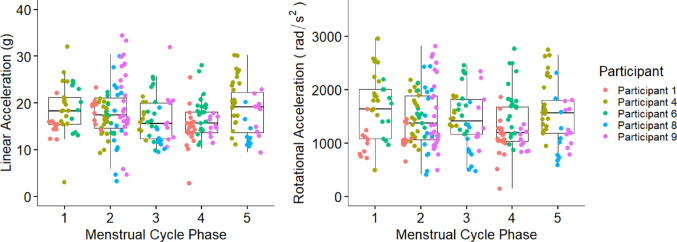
Participant-specific and group mean linear accelerations (*g*) and rotational acceleration (rad/s^2^) for each phase of the menstrual cycle (1 = Menstruation, 2 = Late follicular phase, 3 = Ovulation, 4 = Mid Luteal, 5 = Late Luteal). Box whiskers represent group range. There were no significant differences between phases

256 head impacts (v_ball_ = 6.1 ± 0.5 m/s; a_linear_ = 17.5 ± 5.4 g; a_rotational_ = 1479 ± 548 rad*/s*^*2*^) (see Table 2) were recorded during testing. Analysis revealed no significant differences in peak log a_linear_ (*p* = 0.30) or log a_rotational_ (*p* = 0.25) between menstrual cycle phases (Fig. 2).

**Table 2:** Overview of intensity and volume impact metrics overall and broken down by the 5 phases of the menstrual cycle

Menstrual cycle phase	No. of heading drills recorded (N)	Impacts (N)	Peak linear acceleration (g)	Maximum linear acceleration (g)	Peak rotational acceleration (rad/s^2^)	Maximum rotational Acceleration (rad/s^2^)	Linear contact load (AU)
Menstruation	3	37	18.3 ± 5.2	32.1	1617 ± 611	2960	573 ± 196
Late follicular	8	80	18.2 ± 6.2	34.5	1471 ± 549	2815	486 ± 64
Ovulation	4	44	16.5 ± 4.9	32.0	1480 ± 514	2457	398 ± 104
Mid luteal	5	51	16.1 ± 4.2	28.1	1341 ± 509	2771	460 ± 68
Late luteal	4	44	18.5 ± 5.6	30.3	1535 ± 554	2749	525 ± 114
Average overall	24 (Total)	256	17.5 ± 5.4	–	1479 ± 548	–	500 ± 99

Linear contact load is defined as the sum of z-scored linear and rotational accelerations

### Cognition

#### Reaction times

**Fig. 3:**
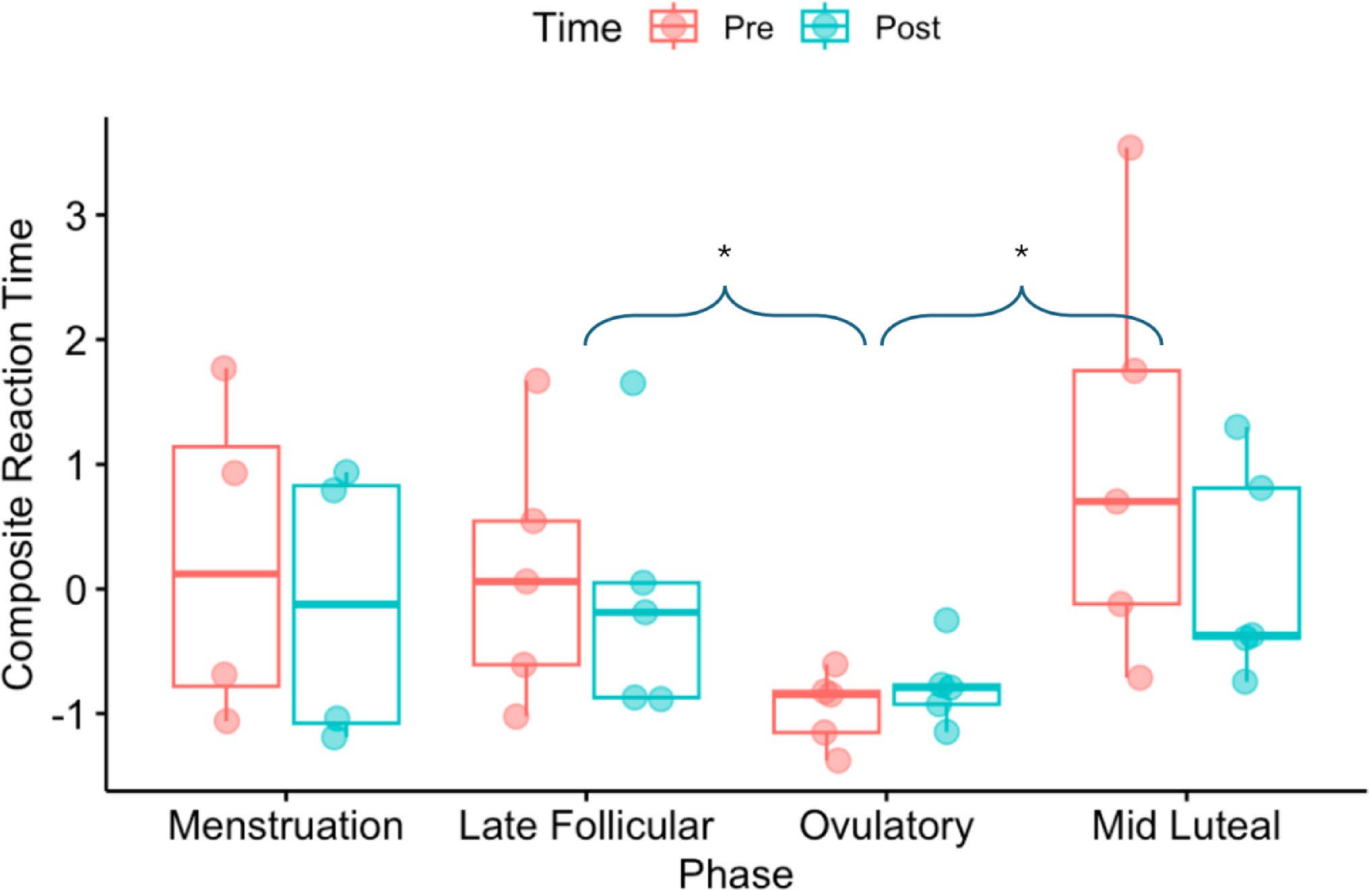
Participant-specific and group mean Composite Reaction Time scores presented during each phase of interest and before (red) and after (blue) the heading drill. Box whiskers represent group range. Significant differences between phases are highlighted by *. There were no significant pre or post heading session differences in performance

Results of Composite Reaction Time produced a main effect of phase, *F*(3,26) = 6.45, p = 0.002, where RTs were shorter during ovulation (CI = -1.83 – 0.14) compared to the late follicular (CI = -0.91 – 1.06) and mid luteal (CI = -0.44 – 1.52) phases (ps < 0.04). No other main effects or interactions were present. Heading had no effect on pre-to-post changes in RT in this small sample (Fig. 3). When analysing individual cognitive tasks, results demonstrated a main effect of phase during the sustained attention and inhibition tasks, *Fs*(3,26) > 6.09, ps < 0.003, respectively. Figure 3 demonstrates that sustained attention and inhibition RTs were shorter during ovulation (CIs = 307 – 585; 309 – 583) compared to the mid luteal phase, respectively (CIs = 510 – 789; 500 – 774) (ps < 0.002), and inhibition RTs were also shorter during ovulation (CI = 309 – 583) compared to the late follicular phase (CI = 436 – 710) (*p* = 0.04). No effects or interactions were present after our analysis of variability of RT.

#### Errors

**Fig. 4:**
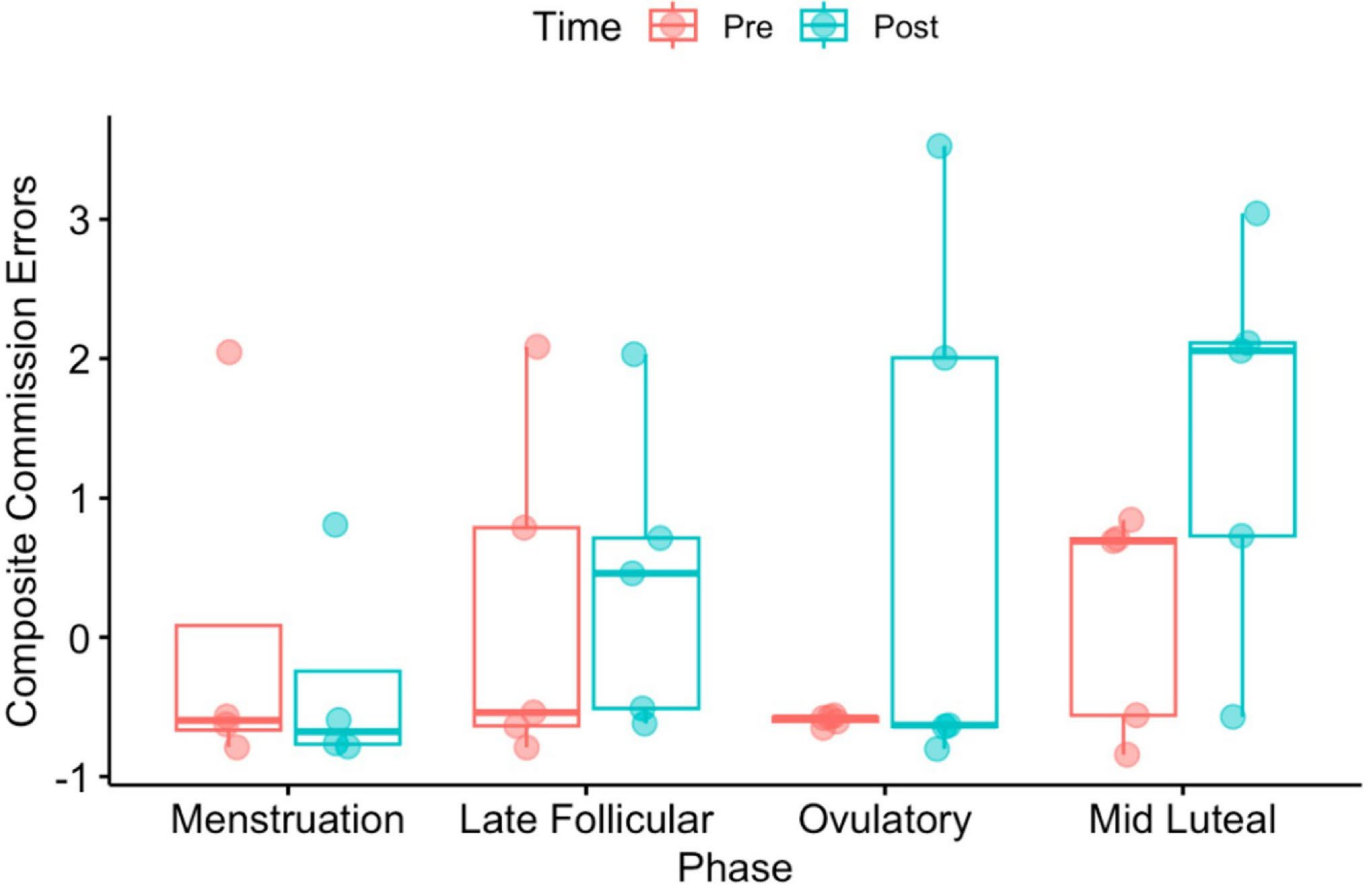
Participant-specific and group mean Composite Commission Errors presented during each phase of interest and before (red) and after (blue) the heading drill. Box whiskers represent group range. There were no significant pre or post heading session differences in performance

Results for total commission errors did not yield any time or phase main effects, nor their interaction, *Fs*(1,26) < 2.74, ps > 0.11 (Fig. 4). Similarly, analysis of timing error did not produce any main effects nor a phase by time interaction, *Fs*(3,26) < 0.55, ps > 0.54. As above, heading had no effect on pre-to-post changes in errors in this small sample.

#### Effects of head acceleration on simple reaction time task performance

**Fig. 5:**
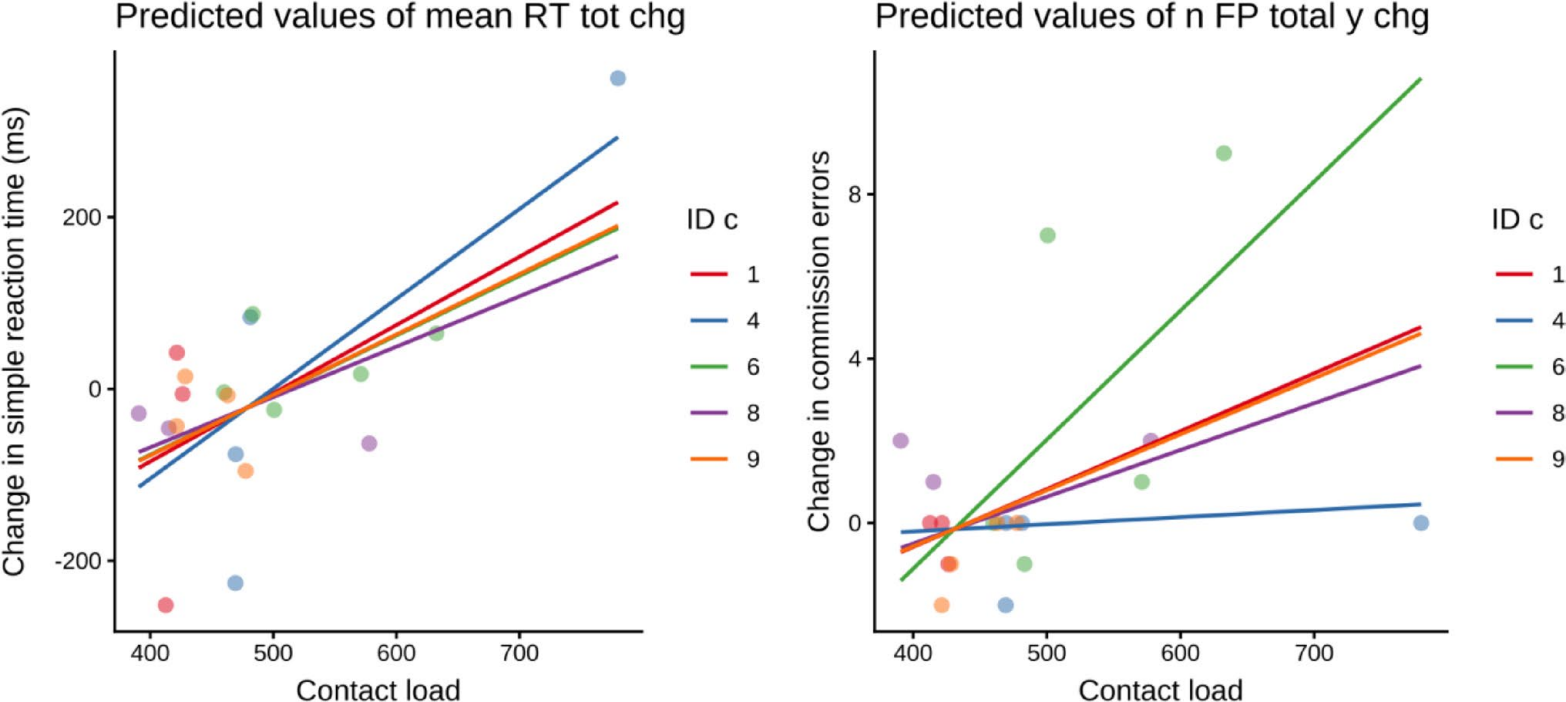
Predicted changes in performance on the simple reaction time task post-heading based on a linear mixed model with random effects. Lines represent slopes of best fit and the coloured dots represent individual participant data points. The average contact load of 10 headers was used to predict pre-post header changes

Contact load (i.e., sum of z-scored a_linear_ and a_rotational_) was assessed via a within-subject predictive model where contact load was found to predict longer simple reaction time task RTs (R^2^ = 0.88, *p* = 0.003; CI = 0.36 – 1.40) but not total commission errors (R^2^ = 0.01, *p* = 0.20; CI = -0.01 – 0.04) immediately after headers. Random effects were assessed per participant for repeated measures. Due to the small study sample, multiple predictors were not included in this model (Fig. 5).

## Discussion and implications

The above work is a proof-of-concept study which aims to describe how multiple non-concussive headers might interact with menstrual phase to affect head acceleration and cognitive function in a sample of elite female footballers. Results revealed that although head accelerations do not differ according to phase, cognition was related to both heading and menstrual phase. We address these preliminary results below before discussing some potential implications for athletes and future directions for the field.

### Menstrual phase does not influence head acceleration

Our analyses revealed no statistical difference in head accelerations between menstrual cycle phases and rejects our hypothesis which suggested that these effects would be strongest during the luteal phase where progesterone peaks. In fact, trends in acceleration appeared to be lower during this phase; however, due to the small sample size recruited during this proof-of-concept study, we cannot sufficiently comment on this result. The heading drill used here was selected based on the finding that 80% of heading in elite female football training was from ‘short passes’ (Bentley et al. 2022). Accordingly, the selected drill was reflective of typical heading seen within training, and *not* during play. In-play metrics recorded from a sample of university-intramural-level male footballers found that heading goal kicks and long passes induced more pronounced acceleration than short passes (Sokol-Randell et al. 2023) and demonstrates the need for further research of head impacts resulting from match-specific scenarios. In terms of head accelerations observed during heading, previous findings have suggested that females are exposed to greater head-impact related accelerations than males (Tierney et al. 2008; Caccese et al. 2018; Mooney et al. 2020; Basinas et al. 2022). However, a recent investigation of a sample of university-level male footballers in a lab-based environment found linear acceleration (26 ± 7.9 g) and angular acceleration (1730 ± 611 rad/s^2^) were similar to those shown here (Barnes-Wood et al. 2024) and indicates the need for further research to optimise protocol delivery and reduce measurement error in in-game settings with a larger sample size.

We acknowledge that this interpretation requires further study with a larger sample size to better understand the factors that might influence head accelerations and, consequently, potential head injury. However, our results have demonstrated the safety and efficacy of the mouthguard equipment employed here, as well as the ease with which the devices can be employed for large-scale SRC monitoring in football. The utility of these devices can be implemented in additional sporting contexts where SRCs and non-concussive injuries are prevalent (e.g., rugby, boxing, American football).

### Heading and menstrual cycle phase appears to influence cognitive function

Overall, we observed shorter and longer post-heading RTs during ovulation and during the luteal phase, respectively. Moreover, headers led to slower simple RTs immediately following 10 headers. The latter effects were particularly pronounced during ovulation and is a result that suggests a potential phase-dependent vulnerability to cognitive function following non-concussive impacts. These results partially align with previous studies that have reported neuropsychological impairment following non-concussive impacts in footballers (Di Virgilio et al. 2016; Levitch et al. 2018; Bruno and Rutherford 2022). As described in the introduction, worse cognition is often reported in the luteal phase (i.e., elevated oestrogen and progesterone) (Ronca et al. 2025), and may be explained by changes in oestrogen and progesterone levels. The excitatory and inhibitory effects of these hormones on the cerebral cortex have been described in previous research (Shaywitz et al. 1999; Smith et al. 1999, 2002). Given certain methodological constraints as well as the small sample used here, we cannot provide unequivocal evidence for any negative or positive effects of acute training-style headers. We can, however, conclude that the cognitive protocol employed here was well-received by the athletes and simple to execute and should be considered suitable for answering these questions in larger-scale studies.

### Limitations and future work

Our proof-of-concept study offers several important insights into the effects of repeated non-concussive headers for female athletes as well as insights into the suitability of our protocol. However, we acknowledge several methodological limitations which require further study. First, the heading drill used in this study had a linear focus with minimal rotational acceleration imparted to the athlete. Based on the link between rotational acceleration and brain strain (Zhang et al. 2006; Sabet et al. 2008), future research may look to design a controlled heading drill that has a more rotational focus. This would allow for a more nuanced (and match-like) investigation of brain injury metrics in relation to menstrual cycle and cognitive function. The drill used here was similar to training and not match-specific situations where ball speed and subsequent impact force could be considerably increased. Moreover, heading proficiency was not directly quantified. The latter is especially relevant given that player ability/comfort with heading may have a mediating effect on short- and long-term outcomes and should be considered in larger studies. Work which investigates these effects will provide a more complete picture of the cumulative effects of head impacts for female footballers. Second, the methodology adopted for this study was carefully designed to ensure a thorough evaluation of the menstrual cycle phases (Elliott-Sale et al. 2021), and phases of the menstrual cycle were identified and tracked via self-report and hormone testing. However, precise, daily hormone tracking was not included in this protocol, nor were we able to measure progesterone levels for accurate confirmation of ovulation (Noordhof et al. 2024; Elliott-Sale et al. 2025). Given we did not enquire about participants’ contraceptive use, it may also be possible that the “regular menstrual cycle” reported by participants was mis-identified or altered by these factors. As well, because of athlete schedules and time commitments, it was not always possible for strict monitoring to occur. Third, the equipment employed here comes with flaws that need to be addressed. For example, given the wide FA-regulation range of the footballs employed here, it may be possible that participants were exposed to varied kinematic effects and therefore experience differential outcomes (England et al. 2025). As well, there is the potential for inaccurate conclusions to be drawn from head impact data. False negatives and positives, however, were largely negated due to the use of video verification. Mouthguard fit was ensured due to custom fitting of guards; however, there has been more recent research regarding ‘decoupling’ of the mouthguard from teeth during an impact (Gellner et al. 2024). We note that poor coupling can increase the percentage error of kinematic measures during an impact, however this noise is typically represented in frequency ranges that either are not present in low magnitude impacts (such as those experienced here) or these frequencies are effectively filtered out due to post-processing. Fourth, the number of headers completed by our participants reflects the athletes’ environment including schedules and other logistical changes. As well, threshold settings for quantifying a header were set at 5 g, so some impacts may not have been captured as a heading event. Video verification mitigated the chances of over/under reporting the headers; however, there was still the possibility of camera occlusion which could have negated this option. Last, because of the inherent difficulty of working with an elite athlete population, the sample size is small. This limits the generalizability of our results and reduces our ability to interpret our findings. Moreover, the confidence intervals reported here reflect a degree of uncertainty in terms of the magnitude and direction of our effects that should be addressed with larger samples and additional measurements where possible. The findings of this proof-of-concept strongly suggest that these outcomes require further investigation with a larger and more robust study which includes accurate serum testing. Future research to expand on this area should include a larger cohort, a sample which includes both sexes, the inclusion of headers which require rotational forces, and a more accurate exploration of hormonal fluctuations (e.g., via advanced biochemical and blood analyses). Although difficult to monitor in this population, it may be possible to eventually include similar tests such as those described above within regular player monitoring activities. The incorporation of these measures and results might help to define individual predispositions to poorer outcomes related to non-concussive head injury, and to inform evidence-based guidelines for injury prevention and management in elite sports. Wider considerations of such work may include educational initiatives targeting athletes, coaches, and healthcare providers, as well as empowering athletes to track their menstrual cycles and communicate any relevant changes to facilitate informed decision-making regarding training, injury prevention, and management. This project should be the first in a wider programme of study to investigate and facilitate long-term athlete-health safeguarding.

## Conclusion

This proof-of-concept study found no statistically significant differences in head accelerations across menstrual cycle phases; however, menstrual phase and head impacts were associated with cognitive performance. Due to the nature of this study, caution must be exercised in interpreting these findings. Our findings underscore the importance of ongoing research into the impact of headers on neurological health, thereby informing the development of strategies that safeguard the athlete's brain.

## Data Availability

The data will be made available from the authors upon reasonable request.

## Footnotes

1 We note that the study by La Fountaine and colleagues was a prospective, observational study without accurate hormonal profiling and included a small sample size. Thus, direct conclusions regarding the association between injury incidence and/or severity cannot necessarily be drawn.

2 Given that serum progesterone was not measured by this test, we note that it was not possible to identify subtle disturbances in menstrual cycle hormonal fluctuations (Noordhof et al. 2024). This is important given that subtle changes in hormone concentrations can influence cognitive performance and/or injury incidence. Thus, we acknowledge that these results are therefore more indicative of “assumed” rather than “confirmed” menstrual phase.
